# Supplementary material for: A Simple and Efficient CRISPR/Cas9 System Using a Ribonucleoprotein Method for Flammulina filiformis
Source: J Fungi (Basel). 2022 Sep 23;8(10):1000. doi: 10.3390/jof8101000 (PMC9604558; doi:10.3390/jof8101000)
Supplement: Supplementary file 1 [file jof-08-01000-s001.zip › jof-1864897-supplementary.pdf]

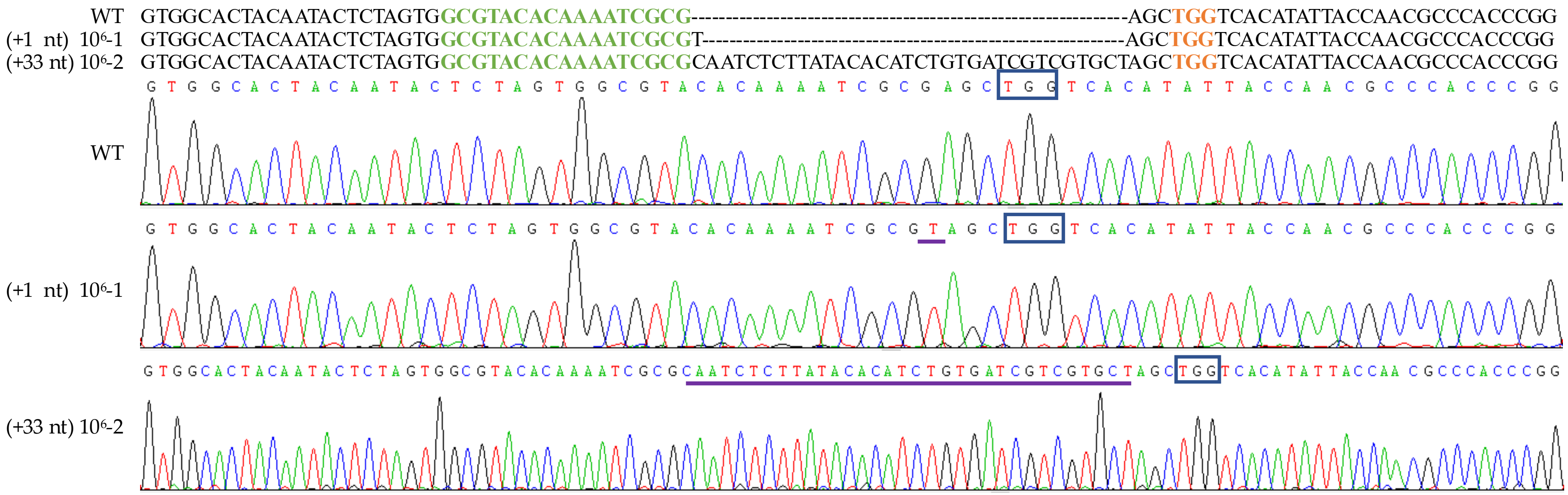

WT GTGGCACTACAATACTCTAGTGGCGTACACAAAATCGCG---------------------------------AGCTGGTCACATATTACCAACGCCCACCCGG
(+1 nt) 10⁶-1 GTGGCACTACAATACTCTAGTGGCGTACACAAAATCGCGT--------------------------------AGCTGGTCACATATTACCAACGCCCACCCGG
(+33 nt) 10⁶-2 GTGGCACTACAATACTCTAGTGGCGTACACAAAATCGCGCAATCTCTTATACACATCTGTGATCGTCGTGCTAGCTGGTCACATATTACCAACGCCCACCCGG
WT
(+1 nt) 10⁶-1
(+33 nt) 10⁶-2

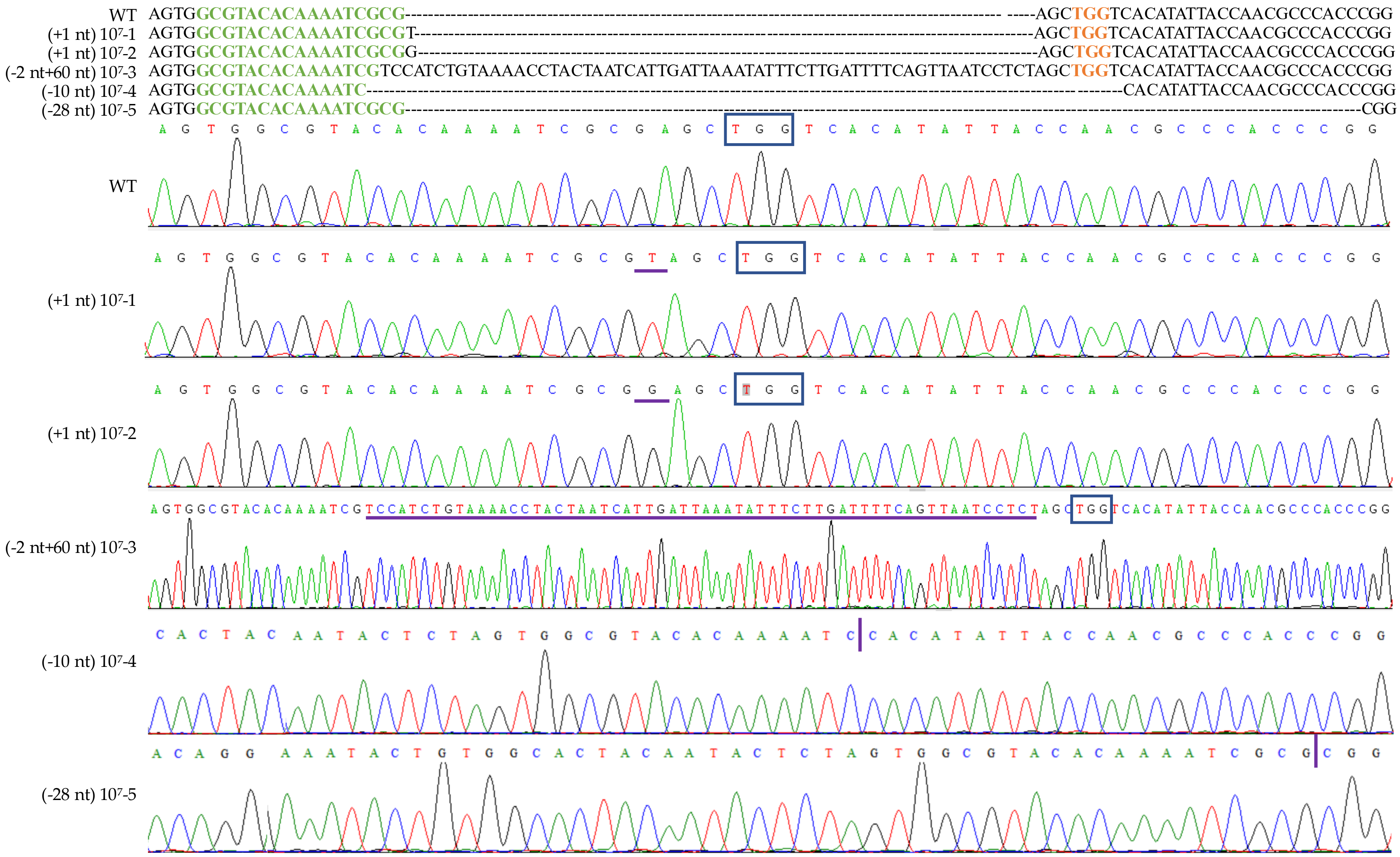

WT AGTGGCGTACACAAAATCGCG------AGCTGGTCACATATTACCAACGCCCACCCGG
(+1 nt) 10^7-1 AGTGGCGTACACAAAATCGCGT------AGCTGGTCACATATTACCAACGCCCACCCGG
(+1 nt) 10^7-2 AGTGGCGTACACAAAATCGCGG------AGCTGGTCACATATTACCAACGCCCACCCGG
(-2 nt+60 nt) 10^7-3 AGTGGCGTACACAAAATCGTCCATCTGTAAAACCTACTAATCATTGATTAAATATTTCTTGATTTTCAGTTAATCCTCTAGCTGGTCACATATTACCAACGCCCACCCGG
(-10 nt) 10^7-4 AGTGGCGTACACAAAATC------CACATATTACCAACGCCCACCCGG
(-28 nt) 10^7-5 AGTGGCGTACACAAAATCGCG------CGG
WT
(+1 nt) 10^7-1
(+1 nt) 10^7-2
(-2 nt+60 nt) 10^7-3
(-10 nt) 10^7-4
(-28 nt) 10^7-5

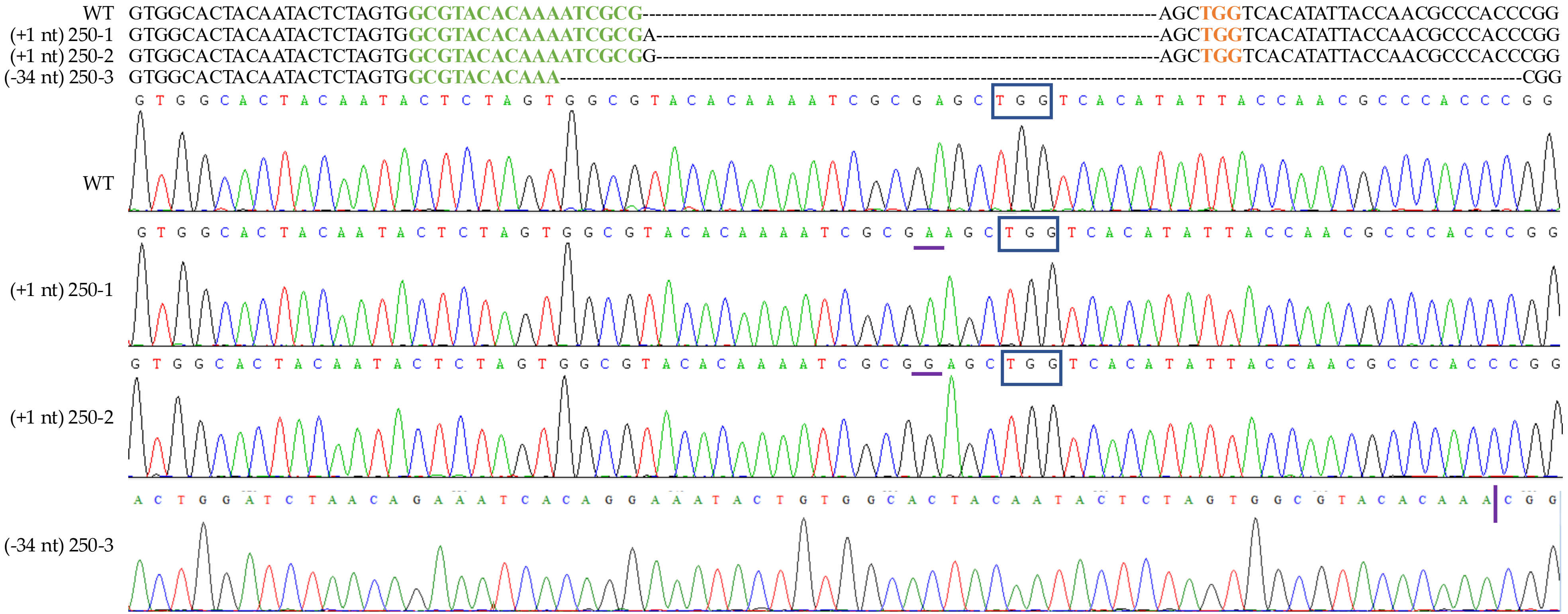
WT GTGGCACTACAATACTCTAGTGGCGTACACAAAATCGCG----------AGCTGGTCACATATTACCAACGCCCACCCGG
(+1 nt) 250-1 GTGGCACTACAATACTCTAGTGGCGTACACAAAATCGCGA----------AGCTGGTCACATATTACCAACGCCCACCCGG
(+1 nt) 250-2 GTGGCACTACAATACTCTAGTGGCGTACACAAAATCGCGG----------AGCTGGTCACATATTACCAACGCCCACCCGG
(-34 nt) 250-3 GTGGCACTACAATACTCTAGTGGCGTACACAAA----------CGG
WT
(+1 nt) 250-1
(+1 nt) 250-2
(-34 nt) 250-3

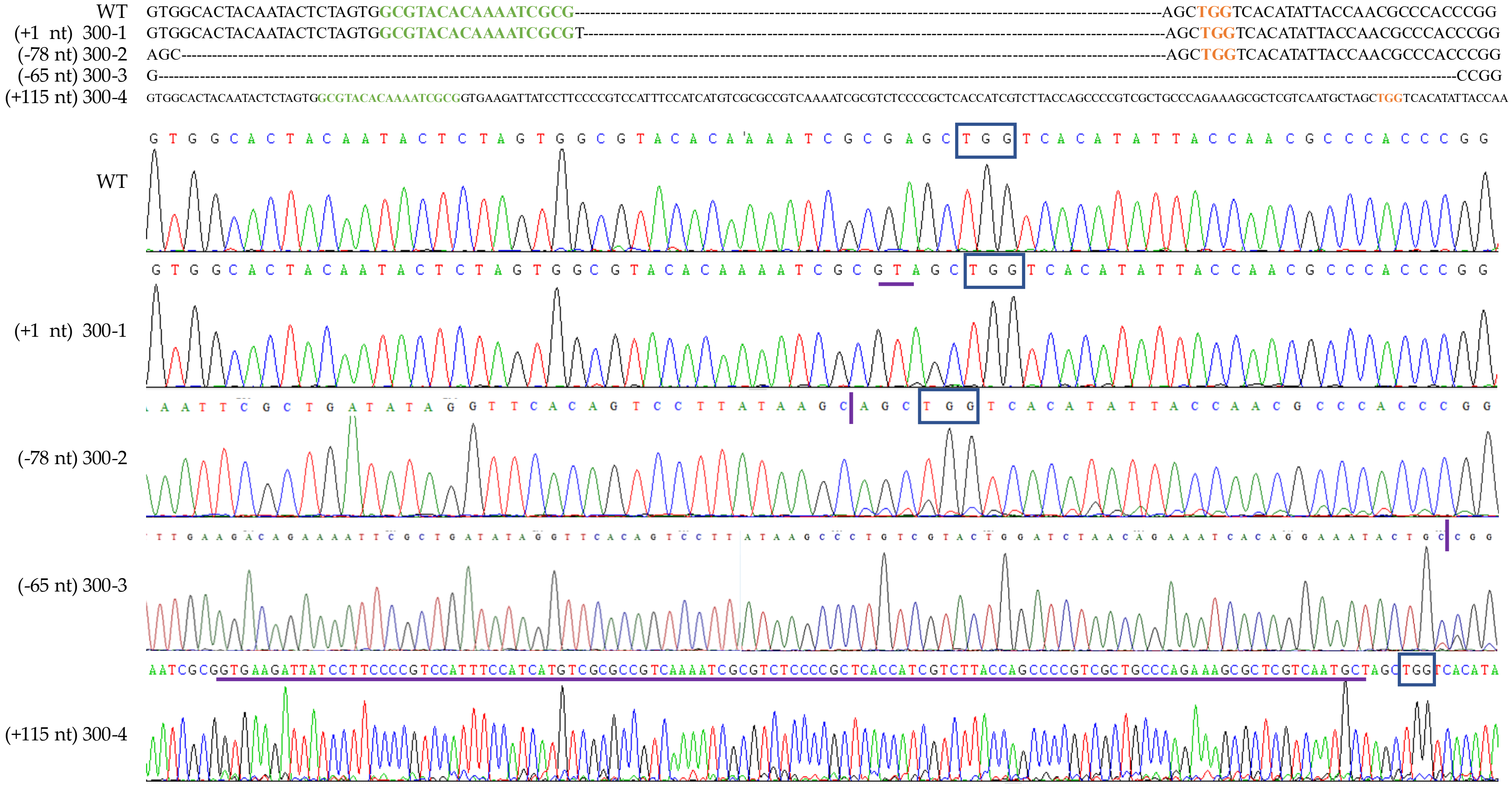

WT GTGGCACTACAATACTCTAGTGGCGTACACAAAATCGCG---AGCTGGTCACATATTACCAACGCCCACCCGG
(+1 nt) 300-1 GTGGCACTACAATACTCTAGTGGCGTACACAAAATCGCGT---AGCTGGTCACATATTACCAACGCCCACCCGG
(-78 nt) 300-2 AGC---AGCTGGTCACATATTACCAACGCCCACCCGG
(-65 nt) 300-3 G---CCGG
(+115 nt) 300-4 GTGGCACTACAATACTCTAGTGGCGTACACAAAATCGCGGTGAAGATTATCCTTCCCCGTCCATTTCCATCATGTCGCGCCGTCAAAATCGCGTCTCCCCGCTCACCATCGTCTTACCAGCCCCGTCGCTGCCCAGAAAGCGCTCGTCAATGCTAGCTGGTCACATATTACCAA
WT
(+1 nt) 300-1
(-78 nt) 300-2
(-65 nt) 300-3
(+115 nt) 300-4

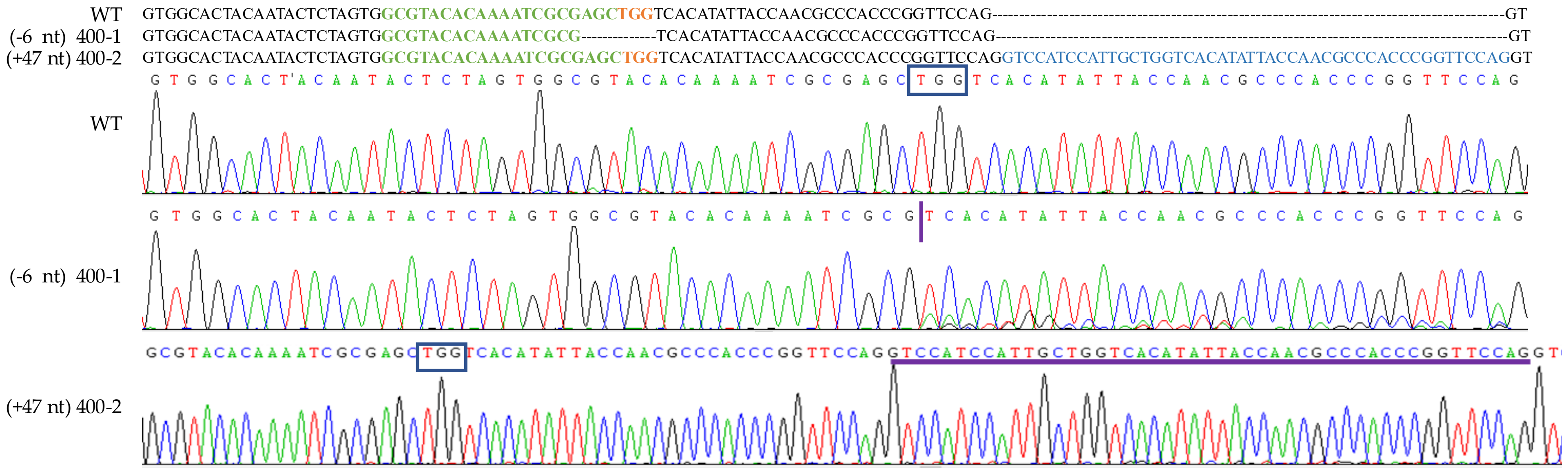

Figure S1. Chromatogram and alignment of pyrG sequences and corresponding mutants.
